# Supplementary material for: Trends in ambulance dispatches related to heat illness from 2010 to 2019: An ecological study
Source: PLoS One. 2022 Nov 7;17(11):e0275641. doi: 10.1371/journal.pone.0275641 (PMC9639828; doi:10.1371/journal.pone.0275641)
Supplement: Attachment — Submitted filename: Reply_to_the_Reviewer_2.docx [file pone.0275641.s001.docx]

Reviewer #2

Trends in ambulance dispatches related to heat illness from 2010 to 2019: An ecological study

Thank you for your very constructive review of our manuscript. Please note that our major revision of the statistical analysis is based on comments from both reviewers, the major revision was as follows:

1. We analyzed the ARDHI data in three groups—All population categories, older people categories (ADRHI_Old), and other than older people categories (ADRHI_YA).

2. To indicate the regional difference in ARDHI, we added the Poisson regression analysis.

3. We added the Fig.6.

All issues you pointed out were all very important. We have made revisions accordingly. Our point-by-point responses are written below each comment. We hope that these revisions will address your concerns.

We highlighted under the line and red font where we modified in the revised manuscript.

Comments by Reviewer #2

*This study examines the relationship between ADHI and WBGT. I believe this is an important topic, with both academic and policy implications. I have the following suggestions to the authors:*

***1. Abstract: State the full name (wet bulb globe temperature) for WBGT in the abstract.***

Ans.

We have expanded the full name accordingly (P2, L38)

【P2, L38】

ADRHI and wet bulb globe temperature (WBGT) data

***2. Methodology: Currently this analysis examines only two variables, ADHI and WBGT. But as the authors' have stated, there are many other factors that influence heat illness. The authors can consider using other statistical methods that can have multiple independent variables (including WBGT and others) and one dependent variable (ADHI), so that we can understand even when other factors are considered or controlled, WBGT still have positive relationship with ADHI.***

***3. other factors to be considered: this is related to point #2. I am wondering if the following factors also influence ADHI: (1) percentages of elderly, percentages of living alone, and/or percentages of elderly living alone. (2) the ratio between numbers of hospitals to population sizes (e.g., how many hosptials per, say, 1 million people). (3) poverty rate. (4) percentages of households/families that have air conditioners. (5) population density. (6) gender.***

Ans. (For the answer Q2 and Q3)

Thank you for your important suggestion. As you pointed out, several factors are related to the incidence of ARDHI, including poverty rate, the ratio between numbers of hospitals to population sizes, or percentages of households/families that have air conditioners. Unfortunately, we did not find these data in each prefecture and every year. We could only find the population data and WBGT data, and these data can be linked to the ARDHI data, which were used in the current study. In addition, we were also interested in the gender difference of ARDHI, but the data source of ARDHI did not include the information on gender. On the other hand, we actually performed multiple regression analysis to investigate factors affecting the occurrence of ARDHI. We used WBGT, Month, and the percentage of elderly people as an independent variable. However, the result was not interesting; WBGT and Month highly affected the ARDHI. If we have data on living situation, living alone or not or use of air condition or not, we can expect an interesting result if we apply multiple regression analysis; but, we could not find these data. Similar to your concern, we acknowledge that providing significant alerting information for heat illness as well as effects of other factors on the occurrence of ARDHI other than meteorological conditions is very important.

***4. Heat awareness influence ADHI: The following paper suggest that heat awareness influence ADHI. Although the authors have listed this as limitations, I think that the authors can still think about how to incorporate this factor in their analysis.***

Ans.

Thank you for your useful comment and suggestion. We have deleted the description on the awareness of “the potential effect of implementing heat illness prevention plans” and added information based on your suggestion (P20, L350-355).

【P20, L350-355】

In addition, other than preventing action for heat illness, previous studies also showed the significance of campaigns for stroke by the National Stroke Foundation of Australia [29]. These preventive actions or campaigns implementation might contribute to having a large impact not only on a decrease in the number of patients or dispatches but also on the awareness for heat illness or stroke.

***5. Lit review: Currently the manuscript does not have good discussion on the factors that influence heat illness or ADHI. I strongly suggest the authors to include this part, so that readers can evaluate the results of this study. For example, I am wondering whether the max daily temperature in one day or the duration of high temperature (high temp that last several days) matter more for heat illness. A proper lit review can clarify the questions.***

Ans.

Thank you for your meaningful suggestion. As you mentioned above, the occurrence of heat illness related to the max daily temperature in one day or the duration of high temperature (high temp that lasts several days) matters more for heat illness, and this hypothesis is supported by several studies. Actually, in the current study, there is a significant relationship between ARDHI and -1day WBGTmax and similar results in the main text. However, because the purpose of the current study was to investigate the relationship between the number of ARDHI and Japanese heat hazard level using WBGT, we could not incorporate your suggestion; rather, we recommend it for future studies.

(P.22 L395-404)

【P.22 L395-404】

Third, the current study indicates that meteorological conditions other than WBGT, including daily maximum or apparent temperature, may increase ARDHI risk; particularly the time lag effect of maximum temperature or average apparent temperature [10]. However, this was because the current study aimed to investigate the relationship between the number of ARDHI and Japanese heat hazard levels based on WBGT. Despite several limitations of the study, our findings provided more realistic WBGT values, and it is believed that the data can help decision makers in making more well-informed decisions about the development and implementation of action plans; this may ensure more effective measures against heat illnesses in Japan.

***6. fig 5: fig 5 suggests that the max temp do not result in most ADHI. That is very interesting, and the authors can elaborate more on this.***

Ans.

Thank you for your comment, we elaborated on this in the main manuscript. (P.17 L.289 to P.18 L.294)

【P.17 L.289 to P.18 L.294】

Interestingly, despite the strong relationships between ARDHI and WBGT (Fig. 4), most ADRHI did not occur in the highest WBGTmax (Fig.6). This corresponds with each of the given WBGT. For example, a total of 2 days were < 35℃ WBGTmax during the study period while 28℃ WBGTmax was observed for 39739 days. Additionally, the ratio of ARDHI per day in each case was 182 (364 cases/2 days) and 8.0 (39739 cases/4958 days).

***7.Fig 9: the analysis suggested that there are no regional differences between regions, but I am wondering how factors listed in point #3 influence the results. For example, how the percentage of elderly influence the results?***

Ans.

Thank you for your constructive comments. First, to clarify the age category effect, we analyzed the ARDHI data in three categories, ADRHI_ALL, ADRHI_Old, and ADRHI_YA.

In addition, as the other reviewer also suggested, we performed Poisson regression analysis to clarify the effect on ARDH by one unit increase of WBGT across each age category. As expected, the result of the Poisson regression analysis shows the difference of exponential β value across each region and age category. This result is shown in Table. 4. (P.16 L.255)

However, the first steep increase in WBGTmax in ARDHI showed no significant difference between ADRHI_Old, and ADRHI_YA in June (P18, L.304-314). We added these very meaningful results in line with your pertinent comment. We appreciate your comments.

【P18, L.304-314】

Conversely, previous studies suggested that Older people are likely to have modest improvement following the heat acclimation program, such as sweat rate response [16] or body fluid regulation [26]. For these reasons, it is likely to the difference in first steep increase WBGT between the age categories, however, that of the WBGT in June was the same between ADRHI_Old and ADRHI_YA at 24℃ WBGTmax. These results suggested that, at least in June, the hazard levels in Japan should be considered based on not being sufficiently prepared to deal with heat stress because of insufficient heat exposure and heat acclimation. Although there were no data regarding heat acclimation in our study, it can be inferred that heat acclimation may be important for preventing heat illnesses, and shifting the hazard level might serve as an early precautionary measure when warm weather is forecasted in early summer.
