# Supplementary material for: Trends in ambulance dispatches related to heat illness from 2010 to 2019: An ecological study
Source: PLoS One. 2022 Nov 7;17(11):e0275641. doi: 10.1371/journal.pone.0275641 (PMC9639828; doi:10.1371/journal.pone.0275641)
Supplement: Attachment — Submitted filename: Reply_to_the_Reviwer_1_final.docx [file pone.0275641.s002.docx]

Reviewer #1

Trends in ambulance dispatches related to heat illness from 2010 to 2019: An ecological study

Thank you again for your careful review of our manuscript. We modified the manuscript based on your comments as indicated below.

***1. The variables' name (i.e. ADRHI_ALL, ADRHI_Old, and ADRHI_YA) can be removed from the abstract.***

***Response***

Per our suggestion, we deleted the variables' names, i.e., ADRHI_ALL, ADRHI_Old, and ADRHI_YA from the abstract.
