# Supplementary material for: Trends in ambulance dispatches related to heat illness from 2010 to 2019: An ecological study
Source: PLoS One. 2022 Nov 7;17(11):e0275641. doi: 10.1371/journal.pone.0275641 (PMC9639828; doi:10.1371/journal.pone.0275641)
Supplement: Attachment — Submitted filename: Reply_to_the_Reviwer_1.docx [file pone.0275641.s003.docx]

Reviewer #1

Trends in ambulance dispatches related to heat illness from 2010 to 2019: An ecological study

Thank you again for your careful review of our manuscript. We modified the manuscript based on your comments as indicated below.

1.　(Old), (YA) can be removed from the abstract.

***Response***

We have removed (Old), (YA) from the abstract.

【Abstract】

Moreover, several studies have reported an age difference in thermoregulation between older and younger adults, implying that the hazard classification should also consider age differences.

2. β value can be replaced by "the coefficients".

***Response***
We have replaced β value with the coefficients in the manuscript. (L.49, 245, 253, 322, 367, 414.)

・Further, Poisson regression analysis revealed that **the exponential of the coefficients** differed（L.49）

・Moreover, the exponential of the coefficients differed in each region and in each age category (Table. 4). （L.243）

・Table. 4 The exponential of the coefficients by each region and age（L.251）

・Additionally, to investigate the effects on ADRHI by one unit increase of WBGT, the exponential of the coefficients was calculated, （L.315）

・　Additionally, further analysis showed differences in the exponential of the coefficients (Table. 4) （L.360）

・Further analysis revealed that the exponential of the coefficients differed in each region and age category. （L.407）

3.　In Table 4: Old means "elderly"? Please change the term and define the age of elderly in the table's footnote.

***Response***

In the text, we have defined “Old” as those aged 65 years old and above, along with the definition for all the age categories. Thus, we have used “Old” in all figures and tables. I hope this is sufficient.
