# Supplementary material for: Trends in ambulance dispatches related to heat illness from 2010 to 2019: An ecological study
Source: PLoS One. 2022 Nov 7;17(11):e0275641. doi: 10.1371/journal.pone.0275641 (PMC9639828; doi:10.1371/journal.pone.0275641)
Supplement: Attachment — Submitted filename: Reply_to_the_Editor_5th.submission_final.docx [file pone.0275641.s004.docx]

Dear Editor,

We wish to re-submit the manuscript titled “Trends in ambulance dispatches related to heat illness from 2010 to 2019: An ecological study”

We deeply thank you for the careful review of our manuscript.

We answer your concerns below;

*Referring to Figure 6, you mentioned that * P<0.05 vs July, August, and September. However, visually the bar represents June looks the same as the bar represents September. I would like to confirm if there is any mistake. Moreover, please also have a check on the same issue on other figures.*

**Answer**

We acknowledge your concern, but we have done statistical analysis using the same way described in the methods section repeatedly, and we have the same result on your concern point. So we did not change figure 6.

*Regarding Figure 7, please add the unit for the x-axis. (i.e. WBGTmax). Please also have a check on the same issue on other figures.*

**Answer**

Thank you for your suggestion, we added the unit at the edge of the x-axis.
