# Supplementary material for: Trends in ambulance dispatches related to heat illness from 2010 to 2019: An ecological study
Source: PLoS One. 2022 Nov 7;17(11):e0275641. doi: 10.1371/journal.pone.0275641 (PMC9639828; doi:10.1371/journal.pone.0275641)
Supplement: Attachment — Submitted filename: Reply_to_the_Editor_6th.docx [file pone.0275641.s005.docx]

Dear Editor:

We wish to re-submit the manuscript titled “Trends in ambulance dispatches related to heat illness from 2010 to 2019: An ecological study.”

We deeply thank you for the careful review of our manuscript. We have made revisions according to your suggestions. Our point-by-point responses are provided below. We hope that these revisions will address your concerns.

The revisions in the main manuscript are indicated using underlined red font.

Thank you for your consideration. I look forward to hearing from you.

Sincerely,

Daisuke Nakamura

Rikkyo Research Institute of Wellness, Rikkyo University

3-34-1 Nishiikebukuro, Toshima-ku, 171-8501 Tokyo, Japan

Email: daisuke.nakamura@ac.cyberhome.ne.jp

Tel and Fax: + 81-050-5575-1772

Q

Regarding the problem in Figure 6, how about you explicitly write down the 95% CI in an appropriate location such as the main text or figure? I think this can make things transparent. Please do the same on other figures with the same

problem.

Answer

We have added the corresponding CI values in the results section as indicated below.

Fig 3 shows the relationship between the number of ADRHI and WBGT_max_. A significant relationship between ADRHI and WBGT_max_ (p < 0.05, r = 0.74, CI: 0.736-0.744, n = 57,336) was observed. The distribution of WBGT_max_ stratified by month and regions are shown in Figs. 4 and 5, respectively. The WBGT_max_ in each month differed significantly, with the differences in all months having differed significantly from other months (p < 0.05, Fig. 4). The WBGT_max_ also differed significantly between regions (p < 0.05, Fig. 5).

Fig 6 shows ADRHI_All stratified by month. It was 0.12 ± 0.20 (CI: 0.117-0.123), 0.67 ± 0.75 (CI: 0.659-0.683), 0.66 ± 0.64 (CI: 0.645-0.666), and 0.12 ± 0.23 (CI: 0.117-0.125) in June, July, August, and September, respectively.
