# Supplementary material for: Trends in ambulance dispatches related to heat illness from 2010 to 2019: An ecological study
Source: PLoS One. 2022 Nov 7;17(11):e0275641. doi: 10.1371/journal.pone.0275641 (PMC9639828; doi:10.1371/journal.pone.0275641)
Supplement: Attachment — Submitted filename: response_to_reviwer_3.docx [file pone.0275641.s006.docx]

Reviewer #3:

Thank you for your review of our manuscript.

We have further modified our manuscript according to your suggestions.

We hope you are satisfied with our response.

To clearly indicate our revisions, the revised parts of the manuscript are in red colored font.

***Q. 1．How was the “first significant increase” defined? Was it through statistical testing?***

Response

Thank you for your comment. We have added a description of the definition of the first significant increase to the manuscript as follows:

Lines 175–177

To compare the differences for the number of ADRHI by month and region, we conducted Kruskal–Wallis test. When a significant difference was detected, the Dann-Bonferroni test was used for post hoc analysis. In addition, to clarify the relationship between ADRHI and WBGT_max_, the WBGT_max_ of the first significant increase in ADRHI compared to the previous WBGT_max_ was defined as the first significant increase point. Moreover, to investigate the effects on ADRHI by one unit increase of WBGT, Poisson regression analysis was performed using SPSS (version 27.0; SPSS Inc, Chicago, IL). The statistical significance was set at p < 0.05.

***Q. 2.
Line 136- June 2010 to September 2019 is 9 years and 122 days.***

Response

We used data from the years 2010, 2011, 2012, 2013, 2014, 2015, 2016, 2017, 2018, and 2019. Hence, we considered the period to be 10 years.

***Q. 3.
Table 1 is not directly relevant to the main result and can be a supplement table.***

Response

As you pointed out, Table 1 is not directly related to the main result; however, this table is very important to understand the results of the study. Because the investigation of this research focused on the regional differences in ADRHI, we think Table 1 is needed to understand the geographical locations of the regions being analyzed. To clarify, each region’s location and the specific location data helps to understand the meteorological conditions, which are very important factors in the occurrence of heatstroke. Thus, we do not think this table should be a supplement. We hope you understand.

***Q. 4.*** ***ADRHI_old and ADRHI_YA- how were they defined? Number of ADRHI in old or young per 100,000 old or young? That is, both the numerator and denominator are limited the subgroup (old or young)?***

Response

Thank you for your comment. We have included definitions of ADRHI_Old and ADRHI_YA in the Methods section as follows:

Lines 168–170

The WBGT was divided per 1℃, from 20℃ to 35℃. The ADRHI was evaluated by absolute value and per 100,000 people (ADRHI_All), per 100,000 people aged 65 years and older (ADRHI_Old), and other than ADRHI_Old (ADRHI_YA).

***Q.5
How was the Poison regression set up? Was ADRHI count used as the response variable? Has the model accounted for the difference in population size for different prefecture? Were separate models fitted for different regions and different age groups? What were the coefficients in Table 4? Coefficient for WBGT as a continuous variable? There be may be curvature in the relationship and WBGT-squared term may also be needed. Were any covariates included? Potential covariates of interest to include are region, the proportion of 65 and above in the population, some measurements of socioeconomic levels of the prefecture, year, month of the year. By fitting all the data in the same model you can perform statistical test to see if the effects of WBGT differ for different regions or differ for different month.***

Response

Thank you for your comment. Before answering this question, we would like to provide an excerpt of the communication between another reviewer, the editor, and us.

Regarding the statistical analysis, we were provided some suggestions by Reviewer #2 during the first peer review process almost 6 months ago. We have attached our responses from that round of revisions below:

Q from Reviewer #2:

***1. In your data, you have daily ADRHI data and also age group information. If you can apply zero-inflated Poisson model on your data, you can know how the effects on ADRHI by one unit increase of WBGT and also identify the effects from different age groups. This contribution will be very large because the heat illness might affect those vulnerable population. The alert signals can be issued to those specific population.***

Response from us:

Thank you for your very constructive comments. As you pointed out, we conducted Poisson regression analysis to investigate the effect on ARDHI by one degree of WBGT across each age category. The result of exponential β value is shown in Table 4, and other parts for the explanation for the analysis.

After the first revision, Reviewer #2 was satisfied with our response. The editor required clarifications in the 2^nd^ review process 5 months ago as follows:

Q from the Editor:

***7. Table 4, 95%CI and significant levels should be given.***

Response from us:

We added the 95%CI in Table 4 in parenthesis.

| Regions | All age categories | Old | Adults | Adolescents | Infants | Newborns |
| --- | --- | --- | --- | --- | --- | --- |
| Hokkaido-Tohoku | 1.43  (1.43–1.43) | 1.42  (1.42–1.43) | 1.46  (1.45–1.47) | 1.39  (1.37–1.40) | 1.31  (1.27–1.35) | NS |
| Kanto | 1.56  (1.56–1.57) | 1.56  (1.55–1.56) | 1.61  (1.60–1.61) | 1.5  (1.49–1.51) | 1.39  (1.36–1.41) | NS |
| Chubu | 1.54  (1.53–1.54) | 1.53  (1.53–1.54) | 1.58  (1.57–1.59) | 1.47  (1.46–1.49) | 1.39  (1.35–1.43) | NS |
| Kansai | 1.56  (1.55–1.56) | 1.56  (1.55–1.57) | 1.59  (1.58–1.60) | 1.47  (1.46–1.49) | 1.44  (1.40–1.48) | NS |
| Chugoku | 1.37  (1.37–1.38) | 1.36  (1.35–1.37) | 1.40  (1.39–1.41) | 1.35  (1.34–1.37) | 1.34  (1.28–1.40) | No-ADRHI |
| Shikoku | 1.55  (1.53–1.56) | 1.53  (1.51–1.54) | 1.60  (1.57–1.62) | 1.50  (1.47–1.53) | 1.37  (1.26–1.48) | No-ADRHI |
| Kyushu-Okinawa | 1.58  (1.57–1.58) | 1.56  (1.55–1.57) | 1.63  (1.62–1.64) | 1.54  (1.52–1.56) | 1.44  (1.38–1.50) | NS |
| ALL | 1.52  (1.52–1.52) | 1.50  (1.57–1.57) | 1.57  (1.57–1.57) | 1.47  (1.46–1.47) | 1.38  (1.37–1.40) | 1.13  (1.02–1.26) |

After the 2^nd^ revision, Reviewer #2 had a few minor comments which were addressed 3 months ago as below:

Q from Reviewer #2:

**2. β value can be replaced by "the coefficients".**

Response from us:
We have replaced β value with the coefficients in the manuscript.

・Further, Poisson regression analysis revealed that **the exponential of the coefficients** differed（Line 46）

・Moreover, the exponential of the coefficients differed in each region and in each age category (Table 4). （Line 242）

・Table 4 The exponential of the coefficients by each region and age（Line 253）

・Additionally, to investigate the effects on ADRHI by one unit increase of WBGT, the exponential of the coefficients was calculated （Line 317）

・　Additionally, further analysis showed differences in the exponential of the coefficients (Table 4) （Line 362）

・Further analysis revealed that the exponential of the coefficients differed in each region and age category. （Line 410）

Response

We hope that these communications have clarified your questions. Please find point-by-point responses to your comments below:

・**Was ADRHI count used as the response variable?**

Yes.

**・Has the model accounted for the difference in population size for different prefecture?**

Yes, we accounted for the population size in the prefectures; however, it was not realistic to collect data from all 47 prefectures. As mentioned above, this study focused on the regional differences in ADRHI; hence, we conducted analysis on each region. Regarding your comment, you proposed that Table 1 be moved to the supplement file; however, to include prefecture difference in the analysis, the information in Table 1 is important. Hence, we have retained Table 1 in the manuscript. We hope you understand our point of view.

**・Were separate models fitted for different regions and different age groups?**

Table 4 presents our analysis of the different regions and age categories.

**・What were the coefficients in Table 4?**

Please refer to the communication with the other reviewer and editor presented above.

**・Potential covariates of interest to include are region, the proportion of 65 and above in the population, some measurements of socioeconomic levels of the prefecture, year, month of the year.**

Thank you for your comment. As mentioned above, this study aimed to investigate data using big data analysis across 10 years to reconsider the Japanese hazard classification for heatstroke in Japan. We acknowledge the importance of the potential covariates recommended by you to be included in the analysis. As you pointed out, the socioeconomic levels of the participants have the potential to affect ADRHI. However, we did not have these data. Similarly, we did not think an analysis by year was suitable for the purpose of this study, as we wanted to address the need to reconsider the Japanese hazard classification for heatstroke based on big data analysis, and not every year’s data.

Again, we thank you for your comments as an expert in statistical analysis. We hope you understand the standing purpose of this study and our point of view. We agree that further analysis, including the potential covariates you pointed out, are required. Accordingly, we have already added this to the limitation section of the Discussion as follows:

(Lines 389–392)

As mentioned, several factors are assumed to be related to ADRHI, such as air pollution, economic status, and health status. However, we could not obtain heat stroke data for this experiment. Addressing this limitation is a concern for future research.

***Q.6
What happened in 2018? Are there explanations for the sudden increase of ADRHI? Is this due to a particular region?***

Answer

Thank you for your comment. We are grateful for your careful review of our data. As you stated, ADRHI in 2018 was higher than in other years. One of the reasons for this was that the average WBGT was higher compared to other years. However, as you know, data were collected from June to September and ADRHI occurred for many reasons. Hence, the exact reason for the sudden increase of ADRHI in 2018 is not known. However, the weather conditions were related to ADRHI significantly; thus, we have added information to the main manuscript to clarify this point.

(Lines 268–269)

To the best of our knowledge, this was the first study to examine the relationship between ADRHI and WBGT in Japan using big data analysis. As expected, the number of ADRHI showed a significant relationship with WBGT_max_ (p < 0.05, r^2^ = 0.55; Fig 3). This result is similar to the previous study, which reported that r^2^ was 0.48 in three big prefectures in Japan [6]. During our experimental period, the average WBGT_max_ was highest in July 2018, while the number of ADRHI cases were highest in 2018.

***Q.7***

***Figure 1. maybe overlay the histogram with a line to indicate the average WBGT across the years to see if the changes correlate?***

Response

Thank you for your suggestion. We have added a line denoting the average WBGT in Fig 1.

***Q.8
Figure 2. what is shown here? the proportion of ADRHI in each age category for each of the 10 years? This figure does not seem to contain much information and there was not much text in the manuscript describing the Figure either. Maybe it should be a supplement?***

Response

Thank you for your comment. As mentioned above, this study aimed to investigate the hazard classification in Japan, and we also wished to analyze the age differences in this context. Therefore, we believe that the proportion of the ADRHI in each age category is one of the main results in the study. It supports the importance of reconsidering the hazard classification based on the age categories. In addition, this study first demonstrated that the age proportion of ADRHI is similar across the years from the point of view of Japan as a whole. In previous studies that focused on heatstroke, the age categories are the most important component, because the ability of thermoregulation of the body is closely related to age. Hence, we think this figure is necessary in the main manuscript, rather than in the supplement.

***Q.9
Figure 3 is very informative and it shows the relationship between ADRH and WBGT. Similar plots showing ADRH in older and younger populations will be helpful.***

Response

Thank you for your suggestion. We were also interested in analyzing the relationships as per your suggestions. Hence, we conducted the analysis; however, the results were not significantly different between age groups. While we agree with your suggestion, we decided to exclude this information from the manuscript as it did not add much to our Discussion, while increasing the word count of our paper.

***Q.10
Figure 4 and 5 are not very informative to me. They only show the distribution of WBGT but the primary interest is the relationship between WBGT and ADRH. It will be more of interest to show ADRH vs WBGT stratified by month and ADRHI_ALL vs WBGT stratified by region instead.***

Response

Thank you for your comment.

In the field of research regarding heatstroke or mortality related to meteorological conditions, the data about the meteorological conditions is the most important to understand the occurrence of heatstroke. Hence, it is generally included in the Results because it is correlated with the incidence of heatstroke. [1-3]

We hope you understand our point of view and hope our response addresses your concerns. Regarding your latter suggestion, we analyzed the data and found that the ADRHI coefficient value was higher in July and August; however, this was related to the number of ADRHI and the range of WBGT compared to June or September. For further clarification, please refer to our response to Q9.

1.	Armstrong BG, Chalabi Z, Fenn B, Hajat S, Kovats S, Milojevic A, et al. Association of mortality with high temperatures in a temperate climate: England and Wales. J Epidemiol Community Health. 2011;65: 340-345. doi: 10.1136/jech.2009.093161. PMID: 20439353.

2.	Ng CF, Ueda K, Ono M, Nitta H, Takami A. Characterizing the effect of summer temperature on heatstroke-related emergency ambulance dispatches in the Kanto area of Japan. Int J Biometeorol. 2014;58: 941-948. doi: 10.1007/s00484-013-0677-4. PMID: 23700200.

3.	Basu R. High ambient temperature and mortality: a review of epidemiologic studies from 2001 to 2008. Environ Health. 2009; 8: 40. doi: 10.1186/1476-069X-8-40. PMID: 19758453.

***Q.11***

***Figure 7. How was the comparison done? Each ADHRI level vs the previous level? Is this how “first significant increase” was defined? Please add the details in the method section.***

Response

Thank you for your comment. Please refer to our response to Q1.

***Q.12
Figure 8 was mentioned in the discussion. The relevant text should be in the result section instead.***

Response

Thank you for your comment. We have moved the text and Fig 8 to the Results section.

(Lines 245–249)

In June, approximately 64.1% of the ADRHI that occurred in the Hokkaido-Tohoku region were related to a WBGT_max_ of > 25°C, which is categorized as “Caution”—the 2nd lowest level in the hazard classification, whereas only 15.0% of ARDHI occurred in the “Caution” level at Kyushu-Okinawa. However, from July to September, most ADRHI occurred at levels over “Warning”.

***Q.13***

***In the*** ***Poisson regression model what if the heat hazard classification is used as a predictor, will there be an interaction between region and heat hazard classification?***

Response

Thank you for your comment. As mentioned in the main text, hazard classification was set regardless of region. On the other hand, the Poisson regression model was used as to clarify the differences between the regions. Thus, we did not check for interactions. As mentioned above, we examined the regional differences and monthly differences using big data in order to help with the reconsideration of Japanese hazard classification. Hence, we believe that investigating the interaction would not have served our purpose. I hope you understand our point of view.
