# Supplementary material for: Trends in ambulance dispatches related to heat illness from 2010 to 2019: An ecological study
Source: PLoS One. 2022 Nov 7;17(11):e0275641. doi: 10.1371/journal.pone.0275641 (PMC9639828; doi:10.1371/journal.pone.0275641)
Supplement: Attachment — Submitted filename: response_to_reviwer_3_Ver.2_final.docx [file pone.0275641.s007.docx]

Reviewer #3:

Thank you for your review of our manuscript. We have gained valuable insights from your suggestions. As per your comments, we have performed Poisson analysis by setting the population size as the offset value; therefore, we used the absolute data for ADRHI in the analysis. Thus, the difference among regions is more clearly indicated, which is supported by another study result mentioned in the manuscript. We are deeply grateful for your expert knowledge on the Poisson regression analysis.

However, regarding question 3, we are unable to adequately understand your concern. Therefore, kindly provide more explanation regarding the insufficiency of our analysis in meeting our study objective and the suggested model.

We apologize for the inconvenience and deeply appreciate your assistance in this regard.

The responses to your comments are presented below and the changes are marked in red font in the manuscript.

**Q. 1．**As the authors listed June 2010 to September 2019 is 10 years (or 10 122-day periods) not 10 years and 122 days.

Response

Thank you for your comment. We have modified the text as follows:

Consequently, the total data set comprised 57,340 ADRHI and contained dispatches from 47 prefectures for 10 years and 122-day period. (L.136)

***Q. 2．***
*Regarding the Poisson regression. All relevant information should show in the manuscript. I still have trouble figuring out how were the models set up. Was separate Poisson model used for each age category and each region combination? The cell for “ALL” and “All age categories” was the relative risk estimate for all data? Were any covariates adjusted for in the model?*

Response

Thank you for your comment. We apologize for the unclear description. We have modified the Methods section to clearly explain the statistical analysis process. (L177-181)

We have used the absolute value in the Poisson model, and the average population was set as the offset value. We hope that these changes address your concern.

Moreover, to investigate the effects on ADRHI by one unit increase of WBGT in each region, Poisson regression analysis was performed using SPSS (version 27.0; SPSS Inc, Chicago, IL). The ADRHI, WBGT_max_, and the average population was set as an objective value, covariates, and the offset value, respectively. The statistical significance was set at p < 0.05.

*Q. 3．
The model with all the data included while adjusting for covariates such as age group, region, month, and potential interaction will be more informative and will help to clarify the relationship between WBGT and ARDHI. This is also consistent with the goal of this study. If there is an interaction between WBGT and month this supports the idea that the same WBGT will influence ARDHI differently for different month, hence the heat hazard classification needs to month specific. Similarly, the comment I made last time “In the Poisson regression model what if the heat hazard classification is used as a predictor, will there be an interaction between region and heat hazard classification?” will help us understand if the heat hazard classification needs to be region specific.*

Response

Thank you for your comment. Unfortunately, we are unable to clearly understand your suggestion. In Question 4, as per your suggestion, we have modified the analysis and the results seem to provide more information regarding the difference among regions for the occurrence of ARDHI. Moreover, we hope that these changes address some of your concerns raised here. Similar to your guidance in Question 4, we would appreciate it if you could suggest specific methods to help us address the issue mentioned here.

In particular, we do not understand the following sentence because the heat hazard classification was not set depending on each region, “will there be an interaction between region and heat hazard classification?” Could you explain this further and suggest methods of analysis to achieve this as you have done in Question 4: “One way to account for the population size is to set the population size of each prefecture as an offset in the Poisson regression model.”

*Q. 4．*
 *The authors mentioned that they “accounted for the population size in the prefect”. Please clarify how was this achieved in the model? It is conceivable that the number of ARDHI will be directly link to the population size. One way to account for the population size is to set the population size of each prefecture as an offset in the Poisson regression model.*

Response

Thank you for your comment, we agree with your opinion that the number of ARDHI will be directly linked to the population size. Thus, in the Poisson model, we performed the analysis by setting the average population size as the offset value. (Table 4)

In addition, according to the modified analysis, we have revised the text describing the Poisson analysis.

*L47*

across each region and age →　across each region

*L245-L246*

Moreover, the exponential of the coefficients differed in each region and in each age category (Table 4).

→　Moreover, the exponential of the coefficients differed for each region ~~and in each age category~~ (Table 4).

*L328*

the exponential of the coefficients was calculated, and the values differed across each age category in each region (Table 4).

　→　the exponential of the coefficients was calculated, and the values differed for ~~each age category in~~ each region (Table 4).

*L420*

Further analysis revealed that the exponential of the coefficients differed in each region and age category.

→　Further analysis revealed that the exponential of the coefficients differed for each region ~~and age category.~~
