# Supplementary material for: Trends in ambulance dispatches related to heat illness from 2010 to 2019: An ecological study
Source: PLoS One. 2022 Nov 7;17(11):e0275641. doi: 10.1371/journal.pone.0275641 (PMC9639828; doi:10.1371/journal.pone.0275641)
Supplement: Attachment — Submitted filename: response_to_reviwer_3_Ver.5_final.docx [file pone.0275641.s008.docx]

Reviewer #3:

Thank you for your thoughtful review of our manuscript. The responses to your comments are presented below and the changes are marked in red font in the manuscript.

*Q1*

*It is not clear how the Poisson regression model was set up. Have region, month, proportion of age over 65 been considered as covariates? They were mentioned in the discussion but not in the method or result. Have likelihood ratio tests been performed to see if each variable is statistically significant? What are the parameter estimates? The newly added paragraph in the discussion belongs to the result and more details about how the statistical modeling was performed need to be provided.*

*What is shown in Table 4? Poisson regression result? Was region the only predictor in the model? Risk estimates from the final Poisson model should be shown instead of a model without adjusting for other covariates.*

Response

Thank you for your helpful comments and suggestions. We apologize that we did not understand your question correctly. We modified the text regarding the Poisson regression analysis in the method and result sections, and revised Table 4. We hope that this modification adequately addresses your concerns.

***Method section*** (p. 176-178)

Moreover, to investigate the effects on ADRHI of a one unit increase in WBGT in each region, a Poisson regression analysis was performed using SPSS (version 27.0; SPSS Inc, Chicago, IL) to investigate the effects on ADRHI of a one unit increase in WBGT in each region. In the Poisson model, the ADRHI was set as the response value, and regions, month, WBGTmax, and proportion of people aged over 65 were set as a predictor. Population size was set as an offset value. The statistical significance was set at p < 0.05.

***Results section*** (p. 239-241)

Moreover, the exponential values of the coefficients differed for each region and month are shown in Table 4 (LRχ2 = 697742.8, p < 0.001). The value of the exponential of the coefficients also differed among regions and months.

***Table 4.***

| **Predictor** | **b** | **SE** | **Waldχ^2^** | **The exponential of the coefficients** |
| --- | --- | --- | --- | --- |
| **Intercept** | **-9.63** | **.03** | **145630.9***** | **< 0.01** |
| **Regions** |  |  | **33902.3***** |  |
| **Ref. value**  **Hokkaido-Tohoku** | **0** |  |  | **1** |
| **Kanto** | **-0.15** | **.01** | **685.7***** | **.859** |
| **Chubu** | **-0.29** | **0.01** | **2536.2***** | **.748** |
| **Kansai** | **-0.19** | **0.01** | **1066.5***** | **.829** |
| **Chugoku** | **-0.45** | **0.01** | **4273.4***** | **.638** |
| **Shikoku** | **-1.02** | **0.01** | **14950.8***** | **.361** |
| **Kyushu-Okinawa** | **-0.75** | **0.01** | **14777.1***** | **.472** |
| **Month** |  |  | **29617.7***** |  |
| **Ref. value**  **September** | **0** |  |  | **1** |
| **June** | **0.64** | **0.01** | **7958.0***** | **1.898** |
| **July** | **0.86** | **0.01** | **24603.3***** | **2.373** |
| **August** | **0.56** | **0.01** | **9878.0***** | **1.748** |
| **WBGTmax** | **0.40** | **0.00** | **280255.3***** | **1.492** |
| **Proportion of people aged over 65** | **-0.01** | **0.00** | **330.2***** | **.992** |

***; P < .001.
