# Supplementary material for: Trends in ambulance dispatches related to heat illness from 2010 to 2019: An ecological study
Source: PLoS One. 2022 Nov 7;17(11):e0275641. doi: 10.1371/journal.pone.0275641 (PMC9639828; doi:10.1371/journal.pone.0275641)
Supplement: Attachment — Submitted filename: response_to_reviwer_3_Ver.6_final.docx [file pone.0275641.s009.docx]

Reviewer #3:

Thank you for your thoughtful review of our manuscript. The responses to your comments are presented below and the changes are marked in red font in the manuscript.

*Q1*

*In a Poisson regression the exponential of the coefficient is the relative risk. Table 4- the current columns are not very helpful for a paper. Maybe only show the point estimate of relative risk and its 95% confidence interval is enough. Given the large sample size, p values do not carry as much information as the 95% confidence intervals.*

Response

Thank you for your kind advice. We modified Table 4. (L252-266)

**Table 4. The Exponential of the Coefficients for Each Region, Month, WBGTmax, and Proportion of People Aged Over 65.**

| **Predictor** | **The exponential of the coefficients** |
| --- | --- |
| **Intercept** | **< 0.01** |
| **Regions** |  |
| **Ref. value**  **Hokkaido-Tohoku** | **1** |
| **Kanto** | **.859 (.848 - .868)** |
| **Chubu** | **.748 (.740 - .757)** |
| **Kansai** | **.829 (.820 - .839)** |
| **Chugoku** | **.638 (.629 - .647)** |
| **Shikoku** | **.361 (.355 - .357)** |
| **Kyushu-Okinawa** | **.472 (.467 - .478)** |
| **Month** |  |
| **Ref. value**  **September** | **1** |
| **June** | **1.898 (1.87 - 1.92)** |
| **July** | **2.373 (2.35 – 2.40)** |
| **August** | **1.748 (1.73 - 1.77)** |
| **WBGTmax** | **1.492 (1.490 - 1.495)** |
| **Proportion of people aged over 65** | **.992 (.991 - .993)** |

The values in parentheses are in the 95% probability range.
