# Supplementary material for: Trends in ambulance dispatches related to heat illness from 2010 to 2019: An ecological study
Source: PLoS One. 2022 Nov 7;17(11):e0275641. doi: 10.1371/journal.pone.0275641 (PMC9639828; doi:10.1371/journal.pone.0275641)
Supplement: Attachment — Submitted filename: response_to_reviwer_3_Ver.7_final.docx [file pone.0275641.s010.docx]

Reviewer #3:

Thank you for your thoughtful review of our manuscript. The responses to your comments are presented below and the changes are marked in red font in the manuscript.

*Q1*

*Please use "relative risk" instead of "exponential of coefficient" for the interpretation of the Poisson model throughout the paper.*

Response

Thank you for your kind advice. We used *"relative risk" instead of "exponential of coefficient" for the interpretation of the Poisson model throughout the paper.*
